# Coagulation profile in bitches with pyometra: Standard tests and thromboelastography

**DOI:** 10.17221/16/2025-VETMED

**Published:** 2025-11-28

**Authors:** Alice Ramesova, Katerina Machackova, Ivana Vanova, Marie Lacinova, Alena Bartoskova, Robert Novotny, Roman Vitasek, Kristina Rehakova, Jaroslav Doubek

**Affiliations:** ^1^Department of Physiology, University of Veterinary Sciences Brno, Brno, Czech Republic; ^2^Small Animal Clinical Laboratory, University of Veterinary Sciences Brno, Brno, Czech Republic; ^3^Department of Reproduction, Small Animal Clinic, University of Veterinary Sciences Brno, Brno, Czech Republic

**Keywords:** antithrombin, disseminated intravascular coagulation, dog, haemostasis, sepsis

## Abstract

This study aimed to determine coagulation changes in bitches with pyometra based on a series of coagulation tests and thromboelastography (TEG), and to assess the incidence of disseminated intravascular coagulation (DIC). Eighteen bitches with pyometra and thirty-four control bitches were examined. Haematological, biochemical, and following haemostasis parameters were measured, including: prothrombin time (PT), activated partial thromboplastin time (aPTT), antithrombin activity (AT), and levels of fibrinogen (FBG), d-dimers (DD), tissue factor (TF), plasminogen (PLG), tissue plasminogen activator inhibitor 1 (TPAI-1), and thromboelastography. DIC was considered present if three or more of these parameters were abnormal: platelet count (<153 × 10^9^/l), PT (>8.1 s), aPTT (>25.5 s), FBG (<0.6 g/l), DD (>0.2 mg/l), and AT (<107%). Significant differences were found in the PT, aPTT, FBG, DD, TPAI-1, clotting time, α-angle, and maximal amplitude. According to our scoring system, two patients were DIC positive. The study found alterations in several coagulation tests and hypercoagulable TEG tracings in bitches with pyometra, which point to excessive activation of coagulation, delayed fibrinolysis, and the presence of DIC. No patient bled abnormally, which may suggest that DIC is not overt in the majority of pyometra patients.

Canine pyometra is a frequent, life-threatening illness characterised by the accumulation of septic exudate in the uterus. This disease typically affects mature intact female dogs during the luteal phase of the oestrous cycle. The pathogenesis involves the effects of oestrogen and progesterone on the endometrium, reduced local immune response, and bacterial colonisation of the uterus. The disease manifests not only locally but also with serious general symptoms and requires immediate surgical or conservative treatment (Smith 2006).

Sepsis, endotoxaemia, and systemic inflammatory response in the afflicted animal can impair haemostasis and, consequently, if not stabilised, can lead to widespread activation of the coagulation system and disseminated intravascular coagulation (DIC). This syndrome manifests as systemic microvascular and macrovascular thrombus formations, which can lead to deficient tissue perfusion and subsequent multiple organ failure, and/or consumptive coagulopathy with life-threatening haemorrhage. DIC is a dynamic process that can be characterised by three phases: the hypercoagulable phase (non-overt DIC), the acute consumptive phase (overt DIC), and the chronic silent phase. Early diagnosis of DIC, together with prompt initiation of therapy, is necessary to increase the patient’s chances of survival (Ho et al. 2005).

Because of these variable stages of DIC, which may arise simultaneously or subsequently in various anatomical areas, no single diagnostic test has been established to detect DIC. Diagnosis is based on the presence of one of the diseases known to be associated with DIC, as well as abnormalities in blood coagulation tests (Goggs et al. 2018). One of the options for identifying the presence of both overt and non-overt DIC is thromboelastography (TEG). This method encompasses not only the soluble components of the coagulation cascade but also the cellular components of haemostasis, thereby providing a nearly complete quantitative evaluation of the global coagulation system (Kol 2012).

Our study aimed to determine changes in coagulation profiles in bitches with pyometra based on routine coagulation tests and measurement of antithrombin (AT), d-dimers (DD), tissue factor (TF), plasminogen (PLG), tissue plasminogen activator inhibitor 1 (TPA-1), and thromboelastography analyses, and to assess the incidence of DIC in bitches with pyometra.

## MATERIAL AND METHODS

### Animals

A total of 52 bitches were enrolled in the study, including 18 client-owned animals hospitalised for pyometra at the authors’ workplace and 34 clinically healthy bitches. Patients with pyometra were selected based on their anamnesis, the presence of characteristic clinical signs, and ultrasonographic examination of the uterus. The diagnosis was confirmed surgically and by a cytological sample obtained from the uterus during surgery. All samples were obtained in accordance with the rules of the Expert Commission for the Protection of Animals.

### Laboratory analyses

Blood samples were taken from the cephalic vein and placed into plastic test tubes (Dispolab, s.r.o., Troubsko, Czech Republic) containing anticoagulants EDTA for complete blood count (CBC), lithium heparin for biochemical analysis, and citrate for coagulation tests and TEG. The samples were processed and measured within 30 minutes. The complete blood count was performed using an impedance haematological analyser CelltacAlpha (Nihon Kohden, Tokyo, Japan). Lithium heparin blood samples were centrifuged for 15 min at 1 000 *g* and analysed on a DPC Konelab 20i biochemistry analyser (Thermo Fisher Scientific, Waltham, MA, USA).

Part of each citrate blood sample was centrifuged for 15 min at 1 000 *g* to obtain plasma and routine coagulation tests: prothrombin time (PT) (Tromboplastin-S; Dialab, s.r.o., Radotín, Czech Republic), activated partial thromboplastin time (aPTT) [(APTT-S; Dialab, s.r.o., Radotín, Czech Republic), (0.025M CaCl_2_; Dr. Kulich Pharma, s.r.o., Hradec Králové, Czech Republic)], and fibrinogen levels (FBG) (Bovinni trombin 100 NIH IU/ml; Dialab, s.r.o., Radotín, Czech Republic) were performed on a two-channel analyser (Coatron M2; Teco, Neufahrn in Niederbayern, Germany).

d-dimer levels were determined in citrated plasma using the NycoCard (Axis-Shield PoC, Oslo, Norway) – a method previously evaluated by Dewhurst et al. (2008). Antithrombin III activity was measured in citrated plasma at the Department of Clinical Haematology, University Hospital, using a chromogenic method (Stachrom ATIII; Diagnostica Stago, s.a.s., Asnières-sur-Seine Cedex, France), a technique previously evaluated by de Laforcade et al. (2008). Tissue factor (coagulation factor III/thromboplastin), plasminogen, and tissue plasminogen activator inhibitor 1 were measured in citrated plasma by quantitative sandwich ELISA using Canine Tissue Factor ELISA Kit (Cusabio, Houston, USA), Plasminogen Dog ELISA Kit (Cusabio, Houston, USA), and Dog Plasminogen Activator Inhibitor 1 (SERPINE1) ELISA Kit (Cusabio, Houston, USA). The absorbance was measured at 450 nm using an ELISA reader (ELX808 Absorbance Microplate Reader; Bio-Tek Instruments, Inc., Vermont, USA), the standard curve was used to determine the concentration of target protein in the samples, and a computer programme Gen5 (RRID:SCR_017317; BioTek Instruments, Inc., Vermont, USA) was used to process ELISA results. If any obtained value was below or above the detection limit of the respective assay, it was replaced with a detection limit value.

Thromboelastographic analysis was conducted using TEG^®^ 5000 Thrombelastograph^®^ Hemostasis System (TEG System; Haemonetics, Boston, USA) using kaolin-coated vial. All samples were examined within 2 h after collection. TEG obtained the following values: reaction time (R-time), clotting time (K-time), α-angle (A), maximal amplitude (MA), and clot lysis at 30 min after maximum clot strength (LY30).

### DIC score

DIC was considered as present if three or more of the following parameters were abnormal: PLT (<153 × 10^9^/l), PT (>8.1 s), aPTT (>25.5 s), FBG (<0.6 g/l), DD (>0.2 mg/l), and AT (<107%). These criteria were modified according to Goggs et al. (2018) and determined based on the reference intervals of the control group. For each parameter (PLT, aPTT, FBG, DD, and AT) outside the reference interval, one point was added to the patient’s DIC score.

### Statistical analysis

The obtained data were analysed for normality using the Shapiro-Wilk test. The results were compared statistically using the Mann-Whitney *U* test. Fisher’s exact test was used to compare the proportion of bitches with anaemia or with DIC between the pyometra and control groups. Linear regression analysis was used to analyse the relationship between HCT and the TEG parameters and to modify the TEG values based on HCT. Nonparametric data were transformed into parametric data to make them suitable for analysis. The incidence rate of DIC in bitches with pyometra was calculated using statistical software. Reference intervals were established for all parameters using a 95% double-sided reference interval. The Tukey test identified outliers. MedCalc statistical software (RRID:SCR_015044; MedCalc Software Bvba, Ostend, Belgium) was used for all calculations. A *P*-value <0.05 was considered statistically significant for all performed analyses.

## RESULTS AND DISCUSSION

Sixteen patients recovered well after the surgical treatment of pyometra, one patient was euthanised during the surgery due to an infaust prognosis and the client’s financial reasons, and one bitch developed renal disease. None of the bitches bled abnormally during surgery. The dogs enrolled in the study were of various breeds [German Shepherd (*n* = 2), American Bulldog, American Cocker Spaniel, Boerboel, Cane Corso, Czechoslovakian Wolfdog, Dachshund, Fox Terrier, French bulldog, Rottweiler, Keeshond, Labrador Retriever, Tibetan Mastiff, Shetland Sheepdog (each *n* = 1), and three crossbred], the mean age was 9.2 years (range 0.7–15.6 years), and the mean weight was 27.8 kg (range 8.0–49.0 kg).

The results of the measurements of selected haematological and biochemical indicators in dogs with pyometra are presented in Tables 1 and 2. Bitches with pyometra frequently exhibited leucocytosis (72%, *n* = 13). However, anaemia was present only in 11.1% (*n* = 2) of the cases. Over 60% (*n* = 11) of patients with pyometra were hyperproteinaemic.

**Table 1:** Blood cell count in bitches with pyometra

Group	Haematological parameter, median, min–max				
	WBC (×10^9^/l)	RBC (×10^12^/l)	HB (g/l)	HCT (l/l)	PLT (×10^9^/l)
Pyometra	21.0	6.7	141.0	45.0	261.0
	9.5–38.2	5.3–8.2	113.0–175.0	34.5–53.0	63.0–512.0
Reference interval	6.0–17.0	5.5–8.5	120.0–180.0	37.0–55.0	200.0–500.0

HB = haemoglobin concentration**;** HCT = haematocrit**;** PLT = platelet count; RBC = red blood cell count; WBC = white blood cell count

**Table 2:** Biochemical profile of bitches with pyometra

Group	Biochemical parameter, median, min–max					
	TP (g/l)	UREA (mmol/l)	CREAT (μmol/l)	ALT (μkat/l)	AST (μkat/l)	ALP (μkat/l)
Pyometra	77.7	4.9	74.5	0.8	0.5	3.0
	43.0–94.0	3.0–10.5	50.2–182.4	0.1–35.4	0.3–30.6	0.6–4.3
Reference interval	55.0–75.0	3.3–8.3	35.0–110.0	0.1–1.0	0.1–1.0	0.1–4.0

ALP* = *alkaline phosphatase; ALT = alanine aminotransferase; AST = aspartate aminotransferase; CREAT*** = ***creatinine; TP = total protein

**Figure 1:**
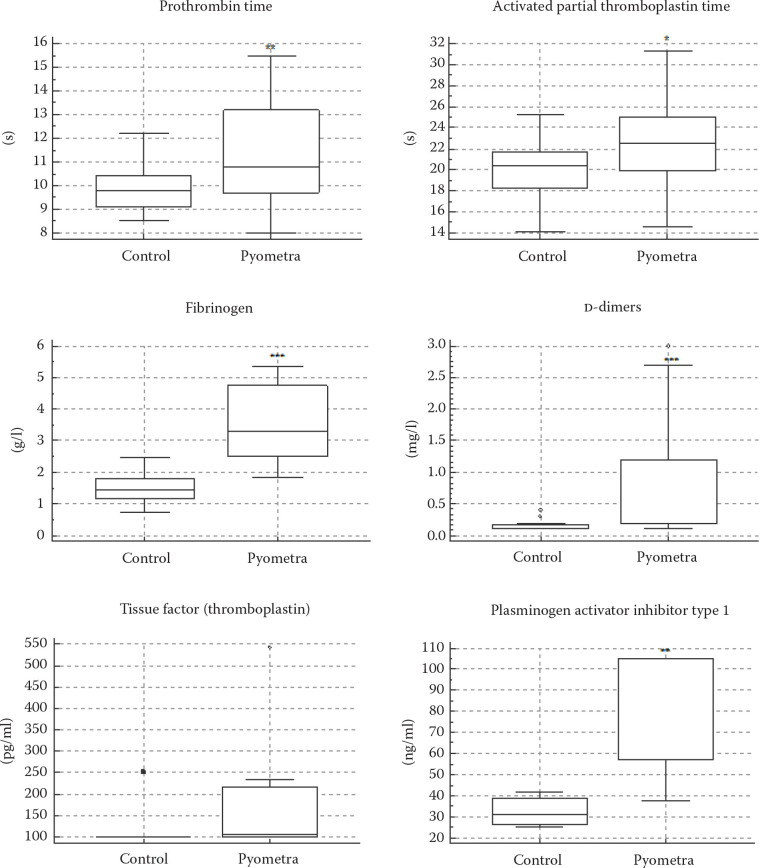
Box plots displaying values of prothrombin time, activated partial thromboplastin time, fibrinogen, d-dimers, tissue factor, and plasminogen activator inhibitor type 1 in healthy control bitches and bitches with pyometra *P < 0.01; **P < 0.05; ***P 0.001 The box shows the values from the lower to upper quartile, and the vertical line inside the box represents the median. The horizontal line stretches from the minimum to the maximum value, excluding outside (circle point) and far-out (black square point) values

Standard coagulation tests and DD, TF, PLG, and TPAI-1 tests were performed in patients with pyometra and control dogs. The results are shown in Table 3. Differences between patients with pyometra and control dogs were found in the following parameters (median patients vs controls): PT (10.8 vs 9.8 s, *P* = 0.009), aPTT (22.5 vs 20.4 s, *P* = 0.014 4), FBG (3.3 vs 1.46 g/l, *P* < 0.000 1), DD (0.2 vs 0.1 mg/l, *P* = 0.000 2), and TPAI-1 (104.9 vs 31.3 pg/ml, *P* = 0.001 3) (Figure 1). Platelet count and antithrombin activity were lower in patients with pyometra. Only 22% of pyometra bitches had decreased PLT (<153 × 10^9^/l), and 11% had reduced AT (<107%).

**Table 3:** Coagulation profile of bitches with pyometra

Group	Coagulation parameter, median, min–max							
	PT (s)	aPTT (s)	FBG (g/l)	DD (mg/l)	AT (%)	TF (pg/ml)	PLG (ng/ml)	TPAI-1 (pg/ml)
Pyometra	10.8**	22.5*	3.3***	0.2***	117.0	106.8	48.8	104.9**
	8.0–15.5	14.6–31.3	1.9–5.4	0.1–3.0	102.0–139.0	100.0–541.4	2.9–79.5	37.5–104.9
Control	9.8	20.4	1.5	0.1	128.0	100.0	37.7	31.3
	8.5–12.2	14.1–25.2	0.73–2.48	0.1–0.4	105.0–138.0	100.0–251.9	2.9–68.3	25.1–41.8

*P < 0.01; **P < 0.05; ***P < 0.001

aPTT = activated partial thromboplastin time; AT = antithrombin activity; D-D = d-dimers; FBG = fibrinogen; PLG = plasminogen; PT = prothrombin time; TF = tissue factor; TPAI-1 = tissue plasminogen activator inhibitor 1

**Figure 2:**
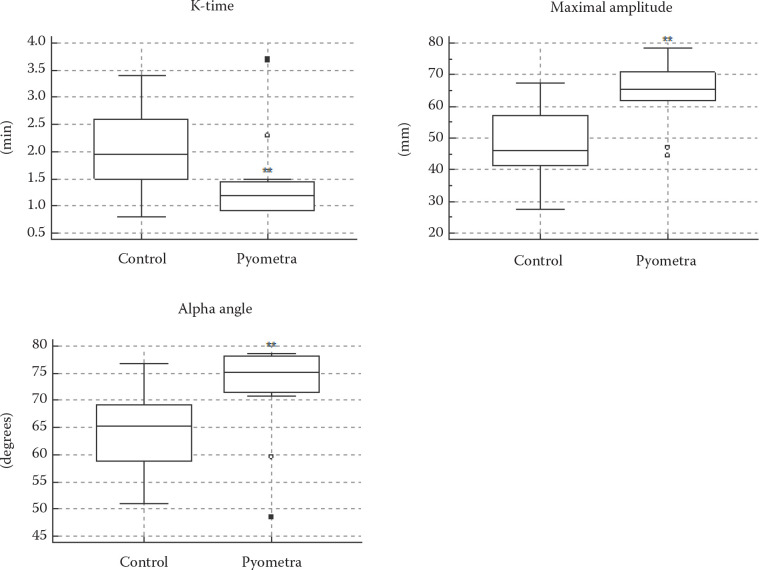
Box plots displaying thromboelastography measures (K time, maximal amplitude, and alpha angle) in healthy control bitches and bitches with pyometra **P < 0.05 The box shows the values from the lower to upper quartile, and the vertical line inside the box represents the median. The horizontal line stretches from the minimum to the maximum value, excluding outside (circle point) and far-out (black square point) values

Obtained TEG results show that hypercoagulation was present in bitches with pyometra (Table 4). Differences were observed in the following indicators, with medians shown (patients vs controls): K (1.2 vs 2.0 s, *P* = 0.006 4), A (75.1 vs 65.5°, *P* = 0.003 8), and MA (65.2 vs 51.8 mm, *P* = 0.000 1) (Figure 2). Changes in K, A, and MA indicators in dogs with pyometra remained statistically significant even after adjusting for HCT (K: *P** = ***0.012 9, A: *P** = ***0.008 9, MA: *P *< 0.000 1).

**Table 4:** Coagulation profile of bitches with pyometra using thromboelastography

Group	Thromboelastography indicator, median, min–max				
	R (min)	K (min)	A (°)	MA (mm)	LY30 (%)
Pyometra	3.2	1.2**	75.1**	65.2**	0.9
	2.3–7.5	0.9–3.7	48.5–78.6	44.4–78.6	0.0–13.6
Control	3.7	2.0	64.7	46.3	7.2
	0.9–5.7	0.8–3.4	39.2–76.9	27.6–67.2	0.0–20.0

**P < 0.05

A= alpha angle; K= K-time; LY 30 = clot lysis at 30 min after maximum clot strength; MA = maximal amplitude; R = R-time

In agreement with the established criteria for DIC scoring, two bitches with pyometra fulfilled three criteria, four bitches fulfilled two criteria, eight bitches fulfilled one criterion, and four bitches fulfilled zero criteria. The incidence rate of DIC among bitches with pyometra was 0.11. The proportion of DIC presence between bitches with pyometra and control dogs was not significant (*P* = 0.115). In comparison to our reference intervals, four patients with pyometra had lower PLT, seven patients had increased PT, and four patients had increased aPTT. None of the patients had lower FBG, and six patients had increased DD. AT activity was lower only in two bitches with pyometra.

This study describes haemostatic changes in bitches with pyometra and the incidence of DIC in these bitches. To our knowledge, no study describing the incidence of DIC in bitches with pyometra has been published so far.

Patients with pyometra enrolled in this study had significantly prolonged PT and aPTT, as well as higher fibrinogen and DD levels. Previous studies have described a considerably lower number of PLTs, higher FBG concentrations, and DD levels (Plavec et al. 2006; Radwinska et al. 2012), prolonged PT and aPTT, and no alteration in AT activity (Radwinska et al. 2012). Our study, by contrast, did not find any significant decrease in platelet count in patients with pyometra, although some bitches (22%) had PLT values lower than the reference interval. Differences in the pre-analytical and analytical phases of laboratory testing may have contributed to the discrepancy between our study and previous studies. Due to generally wide reference ranges for PLT, we believe it would be more accurate to evaluate the decrease in PLT count individually in each patient according to haematologic examination results obtained during regular check-ups. Low numbers or a declining trend of PLT count is a sensitive indicator of extensive systemic activation of coagulation (Levi et al. 1994).

We assume that the prolongation of PT and aPTT was caused by their consumption due to excessive activation of coagulation and the possible induction of DIC (Levi et al. 1994). The tissue damage in pyometra patients can lead to the release of tissue factor, which initiates the activation of the coagulation cascade (Plavec et al. 2006). Simultaneously, clotting factor synthesis may be impaired, as a certain level of cholestasis and hepatocellular necrosis can be present in patients with pyometra (Borresen and Skrede 1980).

Fibrinogen measurement in patients with infection and the consequential systemic inflammation is not very beneficial. Fibrinogen is a positive acute-phase protein and thus is increased during the inflammatory process. High concentrations of fibrinogen in the blood are typically reached as early as 24–48 h after the onset of the infectious process (Bentley et al. 2013; Zapryanova et al. 2013). Despite ongoing consumption during excessive coagulation, fibrinogen levels can remain unaltered for a long time. Also, increased fibrinogen levels can contribute to a hypercoagulable state in septic patients (Bentley et al. 2013).

d-dimer concentrations were significantly increased in bitches with pyometra, which agrees with previously published results (Plavec et al. 2006; Radwinska et al. 2012). Measurement of d-dimer concentration is highly sensitive and the most useful for detecting thromboembolic diseases in dogs (Nelson and Andreasen 2003). Six of our patients with pyometra (33%) had DD above the upper limit of the reference interval, what is less than in a study by Plavec et al. (2006) that found 55% of bitches with increased DD.

Antithrombin activity was lower than the established reference interval only in one patient with pyometra. AT is one of the main thrombin inhibitors, and its levels are rapidly depleted after the onset of DIC (Mesters et al. 1996). Dogs with naturally occurring sepsis exhibit significantly lower AT activity compared to healthy dogs (de Laforcade et al. 2008; Bentley et al. 2013). AT activity is the lowest on the second day after the manifestation of clinical symptoms and increases again from the third day. AT is not only a vital anticoagulant but also shows anti-inflammatory effects (de Laforcade et al. 2008). AT activity, as documented by de Laforcade et al. (2008) and Bentley et al. (2013), was significantly lower than in our patients. Since our patients were probably admitted in different stages of the septic process, it is possible that most of them were in the phase of AT increase.

Levels of TF, PLG, and TPAI-1 were assessed in dogs with pyometra for the first time. We detected a significant rise in TPAI-1 levels and a non-significant drop in the PLG level. The level of TF was not elevated in patients with pyometra. Tissue factor, in complex with factor VII/VIIa, is an essential activator of the coagulation cascade and is expressed by various cells (Pawlinski et al. 2004). A significant increase in TPAI-1 concentration in dogs with pyometra compared to healthy dogs (median, 104.9 vs 31.3 pg/ml) suggests delayed fibrin removal, which may contribute to the development of DIC and subsequent organ ischemia and failure in these patients (Hoshino et al. 2017).

Regarding thromboelastography, the obtained results indicate that bitches with pyometra were largely hypercoagulable, as evidenced by the significantly shortened K-time, wider MA, and higher A values. Several studies have shown the effects of HCT on TEG tracing (Brooks et al. 2014). Our pyometra patients had significantly lower HCT than control dogs (median, 45.0 vs 49.1 l/l), but the drop in HCT values was only mild. Additionally, only two of the eighteen patients were considered anaemic. Thus, we assume HCT values did not affect our TEG results. From biochemical changes, hyperproteinaemia was present in our patients; however, it should not have any effect on thromboelastographic parameters (Crowley et al. 2015).

A previous study evaluated kaolin-activated TEG in bitches with pyometra and detected significant differences in MA, A, and G (clot rigidity) values in these patients (Levi et al. 1994). These results, showing hypercoagulability in pyometra patients, are in accordance with our findings, except for the K value, which was significantly shortened in our patients compared to control dogs, but not in the study by Dorsey et al. (2018). Neither we nor Dorsey et al. (2018) found any bleeding abnormalities in bitches with pyometra. Dogs with naturally occurring sepsis may exhibit standard or hypercoagulable thromboelastography profiles during the first three days after admission to a clinic. K, A, and MA values in these patients are sensitive and specific for distinguishing between dogs that survive and those that do not (Bentley et al. 2013). Another study in dogs described decreased G, A, and MA (hypocoagulation) 4 h after low-dose lipopolysaccharide (LPS) injection (Eralp et al. 2011). Although pyometra is accompanied by LPS-induced endotoxaemia (Jursza-Piotrowska and Siemieniuch 2016), we detected only one pyometra patient with TEG tracings showing hypocoagulability. None of the dogs had increased LY30, and 27% (*n* = 5) of the patients were characterised as normocoagulable, as evidenced by no TEG abnormalities. TEG is a more effective diagnostic tool for early diagnosis of DIC than standard coagulation tests, as it can identify the hypercoagulable DIC stage (Otto et al. 2000).

As pyometra is typically associated with the presence of sepsis and systemic inflammation, the risk of development of DIC is present in these patients. There has been no study so far that addresses the incidence of DIC in pyometra patients. Two patients were classified as DIC-positive according to our scoring system, based on Goggs et al. (2018). The incidence of DIC was 0.11 in our study. This corresponds to the clinical condition of the patients, as none of them showed increased bleeding tendency. The clinical diagnosis of DIC is challenging, particularly in veterinary medicine, as no single diagnostic test exists for DIC. As our patients were admitted in different stages of pyometra and in varying levels of severity of sepsis, it is possible that they were in various phases of DIC development. In the majority of patients, an increased MA was observed, indicating a hypercoagulable state consistent with non-overt DIC. This correlates with the observed low incidence of DIC according to the traditional classification system, which is based on indicators sensitive to overt DIC. In human sepsis, a decrease in MA is associated with the diagnosis of overt DIC (Kim et al. 2020). The authors acknowledge the limitations of this study. Inconsistency in the clinical states of pyometra patients and the different stages of disease development at admission may have caused varying coagulation alterations. Another limitation is the low number of animals included in this study.

In our study, we found alterations in several coagulation tests and hypercoagulable TEG tracings, indicating excessive activation of coagulation and delayed fibrinolysis in pyometra patients. Although none of our patients bled abnormally during the surgical treatment of pyometra, the possible risk of the development of thromboembolic disease should be taken into consideration. Using the DIC scoring system modified to our reference values, we detected 11% of patients with DIC.

Based on the obtained results, we believe that DIC is not overt in the majority of pyometra patients and may remain unnoticed during treatment. Since we do not have any gold standard for determining the diagnostic value of TEG parameters, we can only conclude that the most frequent abnormality observed on TEG was an elevated maximal amplitude.
